# Assessing the Efficacy of Repetitive Transcranial Magnetic Stimulation in the Management of Treatment Resistant Depression: A Scoping Review

**DOI:** 10.1192/j.eurpsy.2023.1773

**Published:** 2023-07-19

**Authors:** M. K. Adu, R. Shalaby, P. Chue, V. Agyapong

**Affiliations:** 1 Psychiatry, Dalhousie University, Halifax NS, Halifax; 2 Psychiatry, University of Alberta, Edmonton, Canada

## Abstract

**Introduction:**

Treatment-resistant depression (TRD) is the failure to accomplish and/or achieve remission after an adequate trial of different classes of antidepressant treatments. TRD presents with significant disability and high prevalence. It results in a substantive socio-economic burden at the community and global levels. TRD. Studies comparing pharmacotherapy and electroconvulsive therapy with repetitive transcranial magnetic stimulation (rTMS) have demonstrated evidence in support of the therapeutic efficacy of rTMS in TRD.

**Objectives:**

This comprehensive scoping review aimed to explore and garner information in the literature regarding the crucial role of rTMS and its therapeutic efficacy as a treatment modality for TRD.

**Methods:**

Electronic data searches in PubMed, PsycINFO, Medline, Embase, and Cinahl were conducted to identify important articles on rTMS for TRD. The data search strategy was limited to articles written in English and published within the last five years, to the date of the data search (February 2022). Articles were reviewed if they reported on a completed randomized controlled trial of rTMS treatment in TRD. Articles were excluded if they were protocols of rTMS on TRD and studies with rTMS for the treatment of conditions other than TRD. The review process was reported using the PRISMA Extension for Scoping Reviews (PRISMA-ScR).

**Results:**

In total, 17 studies met the eligibility criteria for this review. The geographical breakdown of the extracted studies consisted of North America (n = 9), Europe (n = 5), Asia (n = 2), and Australia (n = 1). The frequencies of rTMS applied in the various studies ranged from 5 Hz to 50 Hz, with stimulation intensities ranging from 80% MT to 120% MT. Overall, 16 out of the 17 studies demonstrated evidence that suggested rTMS treatment was effective, safe, and tolerated in the management of patients with TRD.

**Conclusions:**

Overall, rTMS appeard to provide significant therapeutic benefits for patients with TRD through the reduction of depressive symptoms. However, while there is progressive evidence in support of rTMS in TRD, more research is needed to define the standardized protocols of rTMS application in terms of localization, frequency, intensity, and pulse parameters to realize its full potency in TRD.

**Disclosure of Interest:**

None Declared

